# Are Pre‐Hospitalization ECG Abnormalities Associated With Increased Mortality in COVID‐19 Patients? A Quantitative Systematic Literature Review

**DOI:** 10.1111/anec.70016

**Published:** 2024-10-12

**Authors:** Danielle Askey, Ann Smith

**Affiliations:** ^1^ Hazardous Area Response Team Paramedic, South Western Ambulance Service NHS Foundation Trust North Bristol Operations Centre Bristol UK; ^2^ Senior Lecturer in Health Statistics University of the West of England Bristol UK

**Keywords:** COVID‐19, ECG, electrocardiogram, emergency department, mortality, pre‐hospital

## Abstract

**Background:**

While COVID‐19 is predominantly a respiratory disease, cardiovascular complications occur and are associated with worse outcomes. Electrocardiogram (ECG) abnormalities are frequently observed in hospitalized COVID‐19 patients, some of which are associated with increased mortality. It is unclear whether ECG abnormalities occurring before hospitalization are associated with increased mortality. This quantitative systematic literature review aims to determine which ECG changes occurring before hospitalization are associated with mortality and discuss whether these findings can aid the assessment of patients and decision‐making in the pre‐hospital environment.

**Methods:**

A systematic search of the following digital databases was conducted: CINAL, PUBMED, MEDLINE, and Coronavirus Research Database. Eight cohort studies (primary papers) including COVID‐19 patients with ECGs taken in the Emergency Department before hospitalization were selected for quantitative synthesis and results were obtained for the prevalence of ECG changes among survivors compared with non‐survivors. Odds and hazard ratios for ECG abnormalities associated with mortality were also collected and compared.

**Results:**

Identification of ECG abnormalities on pre‐hospitalization ECG is associated with increased mortality in COVID‐19 patients. These ECG abnormalities include non‐sinus rhythm, QTc prolongation, left bundle branch block, axis deviation, atrial fibrillation, atrial flutter, right ventricular strain patterns, ST segment changes, T wave abnormalities, and evidence of left ventricular hypertrophy.

**Conclusion:**

Electrocardiogram assessment in the pre‐hospital environment may be beneficial when assessing COVID‐19 patients and could help identify patients at increased risk of mortality.

## Graphical abstract

Forest Plot displaying statisticallysignificant adjusted odds ratios (aORs) with 95% confidence levels (CIs) forassociation between emergency department electrocardiogram (ECG) findings andmortality. There appears to be an association between some ECG abnormalitiesand mortality in COVID‐19 patients with CIs overlapping, especially in theright ventricular strain pattern where three studies found a statisticallysignificant aOR associated with mortality with CIs overlapping.

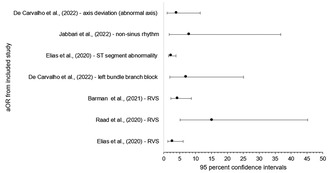

## 1 Introduction

Coronavirus disease (COVID‐19) is a highly transmissible infectious disease caused by severe acute respiratory syndrome coronavirus 2 (SARS‐CoV‐2) originating from and first discovered in Wuhan, China in December 2019 (Peiris et al. 2022). In January 2020, the World Health Organization declared COVID‐19 a public health emergency of international concern; subsequently, infections increased rapidly across the world which combined with the high severity of the disease led to COVID‐19 being declared a global pandemic in March 2020 (Kakodkar, Kaka, and Baig 2020). COVID‐19 continues to be a contributing factor to excess mortality in the United Kingdom (UK) with 619 deaths involving COVID‐19 recorded in the week ending March 17, 2023 (Office for National Statistics 2023). The National Health Service faced significant pressures throughout the pandemic with increased emergency ambulance calls and emergency department attendances (Flynn et al. 2020; Finch and Tinson 2022).

Beyond the respiratory effects of COVID‐19, several known complications are associated with the disease, including; myocardial infarction, myocarditis, arrhythmias, heart failure, Takotsubo cardiomyopathy, vascular dysfunction, and thromboembolic events (Krishna et al. 2024). Cardiovascular involvement contributes to increased mortality (Santoso et al. 2020; The Task Force for the management of COVID‐19 of the European Society of Cardiology 2022) and electrocardiogram (ECG) assessment can assist in the identification of cardiac involvement (Mehraeen et al. 2020; Thakore et al. 2021). Long et al. (2021) explicate the relationship between ECG abnormalities and COVID‐19 within a narrative review which highlights multiple pathophysiological mechanisms including cytokine storm, hypoxic injury, electrolyte abnormalities, plaque rupture, coronary spasm, microthrombi, or direct endothelial or myocardial injury.

Observational studies among hospitalized COVID‐19 patients have indicated that ECG changes are associated with increased mortality (Coromilas et al. 2021; Yuan et al. 2021). A large multicenter cohort study including 2451 adults hospitalized with COVID‐19 by Pinto‐Filho et al. (2023) found that patients with major ECG abnormalities recorded during their hospitalization have a higher risk of 30‐day mortality. This study benefits from a large population size and multicenter methodology including 40 hospitals across 23 countries which increases the validity of results. However, this study included patients who had ECGs taken at any time during their hospitalization and therefore lacks external validity to patients assessed in the pre‐hospital environment.

A systematic review and meta‐analysis by Garcia‐Zamora et al. (2021) found that ECG changes including at QTc prolongation, ST‐segment deviation, and cardiac arrhythmias were observed in COVID‐19 patients during hospitalization and associated with worse outcomes. A similar systematic review and meta‐analysis by Wawrzeńczyk and Grabowski (2022) concluded that ECG assessment as part of the triage of COVID‐19 patients can assist prognostication as they found an association between prolonged QRS, increased QTc interval, and increased heart rate and poor outcomes. These studies are likely to be impacted by cofounding variables associated with hospitalization such as medication administration. A systematic review by Mehraeen et al. (2020) found that medication‐induced ECG changes such as QTc interval prolongation are associated with medications that may be administered to COVID‐19 patients such as chloroquine, hydroxychloroquine, and azithromycin.

It remains unclear whether ECG changes occurring before the patient receives in‐hospital treatment are associated with increased mortality. Understanding whether there is an association could help healthcare professionals assessing patients within the pre‐hospital environment to manage risk by identifying patients in danger of deterioration therefore ensuring those who need ongoing monitoring and treatment are accurately identified. There is a gap in the research relating to ECG abnormalities occurring in COVID‐19 patients before hospitalization; therefore, this quantitative systematic literature review aims to investigate which ECG abnormalities occurring before hospitalization are associated with mortality in COVID‐19 patients.

### 1.1 Objectives

The primary objective of this literature review is to interpret and appraise the current evidence surrounding early ECG changes in COVID‐19 and their association with mortality. The secondary objective is to discuss the significance and implications of these findings to healthcare professionals assessing patients with COVID‐19 in the pre‐hospital environment.

## 2 Methods

### 2.1 Search Strategy

The following databases were searched: CINAL, PUBMED, MEDLINE, Coronavirus Research Database, and the COCHRANE library. These were searched using the search strategy shown in Table 1.

**TABLE 1:** Search strategy.

Search criteria 1	“COVID 19” OR “COVID‐19” OR “coronavirus” OR “SARS‐CoV‐2” OR “coronavirus disease 2019 OR “severe acute respiratory syndrome coronavirus 2” OR “coronavirus disease 19”
AND	
Search criteria 2	“ECG” OR Electrocardiogra*” OR “ECG abnormalities” OR “Abnormal ECG” OR “ECG findings” OR “electrocardiogra* findings” OR “abnormal electrocardiogra*” OR “electrocardiogram abnormalities” OR “pathologic* electrocardiogra*” OR “pathologic* ECG”
AND	
Search criteria 3	3. “Adverse outcomes” OR “mortality” OR “morbidity” OR “ICU” OR “Intensive Care Unit” OR “critically unwell” OR “Critical Care Unit” OR “critically ill” “Prognos*” OR “predict*”

Results were filtered to include articles with publication dates within the past 5 years and availability in English language and full text. A supplementary search of the World Wide Web using the Google search engine with the terms “Emergency Department ECG changes COVID 19” was also undertaken, as was a search of the reference list of included studies. The sources were last searched on 30/05/2023.

### 2.2 Selection Process

Studies investigating ECG changes in COVID‐19 patients met the inclusion criteria providing that ECGs were taken early, with “early” considered as ECGs taken prior to hospitalization, for example, on initial assessment in the Emergency Department (ED) or before in‐hospital treatment is initiated. The rationale for this is that on initial scoping of the literature, no pre‐hospital studies were found; the next patient group representative of the pre‐hospital patients are patients presenting to the ED, prior to receiving any in‐hospital interventions.

Studies focusing on ECG changes occurring during hospitalization following ED presentation or admission were excluded because these patients are unlikely to be representative of the patients seen in the pre‐hospital environment and will likely only include only severe cases of COVID‐19. This would introduce confounding variables such as in‐hospital interventions including medication administration which may impact external validity of results to pre‐hospital care.

Study titles were examined for suitability based on the inclusion and exclusion criteria defined in Table 2. The abstracts and full text for studies not excluded by title were then further screened for relevance by ensuring within the full text that mortality was used as a primary outcome measure and that ECGs were taken at ED presentation or ED admission. Similar literature reviews with different inclusion criteria and outcome measures will be considered within the discussion to assist interpretation of results.

**TABLE 2:** Inclusion and exclusion criteria.

Criteria	Inclusion	Exclusion
Population	Patients testing positive for COVID‐19 Adult patients ≥ 18 years old ECG taken in ED or on ED admission	Children < 18 years old Unclear when included ECGs were taken ECG is taken during hospitalization or on admission to the ward or intensive care unit
Outcome measure	Used mortality as at least one of the outcome measures	Did not use mortality as an outcome measure
Methodology	Prospective or retrospective observational cohort studies	Case studies or case reports Expert opinion Literature reviews (excluded from quantitative synthesis but included within discussion)
Dates	Limited to the past 5 years	Publication over 5 years ago
Language	Studies in English language	Study not available in English language
Availability	Available in full text	Not available in full text
		Duplicates were excluded

### 2.3 Data Collection

A single reviewer worked independently to collect data from each study. Variables for which data were sought from included studies comprised: study objective, population size, mean age (±standard deviation (SD)), sex, study methodology, location, single‐ or multicentered study, when ECGs were taken, primary outcome measures, and ethical approval. The data were tabulated. Assumptions made about identified missing or unclear data were dependent on the type of data missing and considered on an individual basis.

### 2.4 Outcome Measures

Studies were examined for the different ECG changes and outcome measures that they assessed and results from studies were compared. Effect measures for which data were collected included adjusted odds ratios (aOR) or hazard ratios (aHR) for in‐hospital mortality produced from the included studies multivariate logistic regression analysis which will be collected for each ECG change that the studies included. Unadjusted ORs or HRs were not included to minimize the effect of confounding bias associated with confounding variables affecting mortality. 95% confidence intervals (CI) and *p* values were obtained with values < 0.05 accepted as statistically significant. For studies that did not calculate aORs or aHRs, the percentage of prevalence of each ECG change in survivor and non‐survivor groups was collected along with *p* values. Full results of collected data were tabulated and are available in Appendix S1.

### 2.5 Analysis and Synthesis

Tabulated results from data collection were used to present statistically significant aORs in a Forest plot with corresponding 95% CIs. ECG changes with a statistically significant difference in prevalence between surviving and non‐surviving patients were presented within a bar chart. A second bar chart was created displaying the difference in the prevalence of sinus rhythm in survivors compared with non‐survivors. The Forest Plot and two bar graphs were formulated in Microsoft Excel. These methods were used to synthesize results and consolidate the extensive data for ease of interpretation and allow assessment of statistical homogeneity or heterogeneity. Full results of data used in the synthesis of results and formulation of graphs and Forest Plots are available in Appendix S1 in tabulated format.

Where numerical values rather than percentage values were provided within an included study, data conversions of this number to a percentage were undertaken to facilitate the synthesis of results. Missing data were left blank within tables and results where *p* values were unavailable or not statistically significant were not included in result graphs but are available in within Appendix S1.

### 2.6 Risk of Bias Assessment

An individual reviewer used the Clinical Appraisal Skills Programme (CASP) cohort study checklist (CASP 2018) to critically appraise included studies and assess for risk of bias (RoB). Studies were classified as high, moderate, or low‐quality evidence depending on the results of the CASP tool appraisal. Full CASP appraisal results are available in Appendix S1 and a RoB rating for each included study will be presented within the study characteristics table.

### 2.7 Reporting Bias Assessment

Missing results within the synthesis were considered on a case‐by‐case basis. The rationale for missing results identified within the included studies was for the authors’ explanation and where missing data are not explainable or justified this will infer reporting bias which will be highlighted within the results section.

### 2.8 Certainty Assessment

95% CIs were included where available and interpreted to determine whether they increase or decrease certainty in results.

### 2.9 Ethics

Ethical approval not required for this systematic literature review because the data collected were based on already published data with no primary research undertaken. Included studies were assessed for ethical approval and the source of ethical approval was collected and presented in the study characteristics table.

### 2.10 Registration and Protocol

This review was not registered. This review was reported following Preferred Reporting Items for Systematic Reviews and Meta‐Analyses guidelines (Moher et al. 2009).

## 3 Results

### 3.1 Study Selection

**FIGURE 1:**
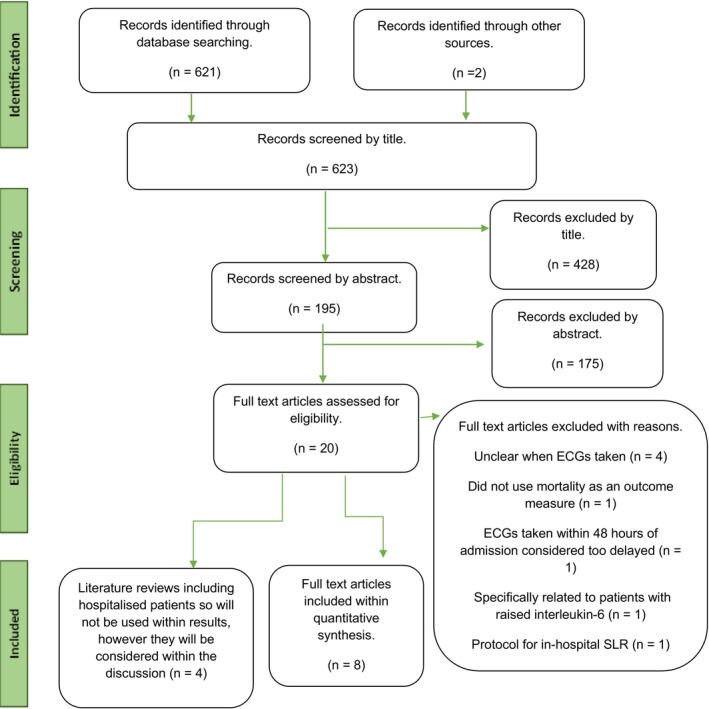
Study selection flow chart (adapted from Page et al. 2021).

The literature review identified 623 studies. Through the application of inclusion and exclusion criteria (see Figure 1), 8 studies were selected for quantitative synthesis (see Table 3) and 4 literature reviews (see Table 4) were included for comparison within discussion.

### 3.2 Included Studies

**TABLE 3:** Study characteristics and risk of bias assessment (full CASP assessment available in Appendix S1; Table S1).

Study	Objective	Type of study	Location	Ethical approval	Included population	ECG taken	Primary outcome measure	Quality of evidence based on RoB rating^a^
Elias et al. (2020)	To study whether combining vital signs and ECG analysis can improve early prognostication	Multicenter observational ‐retrospective cross‐sectional analysis	New York, USA	Approved by the Columbia University Irving Medical Center Institutional Review Board	1258 adult patients Mean age 61.6 (±18.4 years) 44.8% female	In the ED	Mechanical ventilation or death within 48 h of diagnosis	Medium
Jabbari et al. (2023)	To evaluate the association between ECG abnormalities and COVID‐19 clinical outcomes	Single‐center observational—retrospective cross‐sectional analysis	Bandar Abbas, Iran	Approved by the Ethics Committee of Hormozgan University of Medical Sciences	239 adult patients Mean age 55.18 (±16.85 years) 48.3% female	At ED admission	In‐hospital mortality	Low
De Carvalho et al. (2021)	To assess whether specific ECG patterns could be related to in‐hospital mortality in COVID‐19 patients presenting to the ED	Multicenter retrospective study	Three hospitals in France	Approved by Ethics Committee: Groupe Nantais d'Ethique dans le Domaine de la Santé	275 adult patients Mean age was 70 (±16 years) 43% female	At ED admission	In‐hospital mortality	Medium
Raad et al. (2021)	To assess the association of new heart strain patterns on presenting ECG with outcomes of patients hospitalized with COVID‐19	Single‐center retrospective observational cohort study	Detroit, USA	Approved by the Institutional Review Board (#13774)	314 adult patients Mean age 60 (±14 years) 48% female	At a presentation in the ED	Mortality, need for mechanical ventilation, and their composite	High
Barman et al. (2021)	To investigate the prognostic value of right ventricular strain in COVID‐19 patients	Single‐center retrospective observational cohort study	Istanbul, Turkey	Complies with the Declaration of Helsinki. Approved by the local ethics committee	324 adult patients Mean age 64.2 (±14.1 years) 42% female	Patients presenting to the ED, ECG was taken at hospital admission before any treatment was started	In‐hospital mortality	High
Savelloni et al. (2022)	To describe ECG alterations in ED presentation or developed in hospitalization in COVID‐19 patients with mortality	Single‐center retrospective observational cohort study	Italy	Approved by the Ethics Committee of Policlinico Umberto I	190 adult patients Median age 66 (interquartile range 55–80 years) 44% female	On ED admission	28‐day mortality	Medium
Denegri et al. (2021)	To compare clinical and ECG characteristics of patients with COVID‐19 pneumonia	Single center retrospective observational cohort study	Modena, Italy	Unclear	201 adult patients Mean age 68.5 (±14.7 years) 35.8% female	At ED admission	30‐day mortality	Medium
Kunt, Kozaci, and Torun (2021)	To assess parameters associated with mortality in COVID‐19 patients in the ED	Single center prospective observational cohort study	Antalya, Turkey	Approved by the Alanya Alaaddin Keykubat University Faculty of Medicine Ethics Committee. Informed consent was gained	419 adult patients Mean age 51 (±17 years) 39.5% female	At ED admission	In‐hospital mortality	Low

^a^ High‐quality evidence is associated with a low overall risk of bias, medium‐quality evidence is associated with some risk of bias, and low‐quality evidence is associated with a high risk of bias based on judgment following CASP appraisal (see Appendix S1).

**TABLE 4:** Literature reviews included within the discussion.

Study	Title	Objective	ECG taken	Results
Garcia‐Zamora et al. (2021)	Arrhythmias and electrocardiographic findings in Coronavirus disease 2019: A systematic review and meta‐analysis	Examine the association of cardiac arrhythmias and their relationship with adverse outcomes in COVID‐19 patients	Any time during hospitalization	QTc prolongation, ST‐segment deviation, and other forms of cardiac arrhythmias were observed in hospitalized COVID‐19 patients. And associated with a worse prognosis
Wawrzeńczyk and Grabowski (2022)	Evaluation of the prognostic value of the admission ECG in COVID‐19 patients: a meta‐analysis	To assess the prognostic value of the admission of ECG patients	On hospital admission (but included patients with ECGs taken after initiation of in‐hospital treatment including pharmacological treatments)	COVID 19 patients with significantly longer QRS complex, increased QTc interval and greater HR has worse outcomes
Long et al. (2021)	Electrocardiographic manifestations of COVID‐ 19	Narrative review outlining the pathophysiology and electrocardiographic findings associated with COVID‐19	At any time during COVID‐19‐positive patients	COVID‐19 can negatively impact the cardiovascular system through several pathophysiological mechanisms which can lead to abnormal ECG findings
Mehraeen et al. (2020)	A systematic review of ECG findings in patients with COVID‐19	Aim to identify different observed ECG findings in COVID‐19 patients and discuss their clinical significance	Systematic review of case reports, case series, and observational studies of in‐hospital patients	Some included studies reported drug‐induced prolonged QTc interval due to administration of chloroquine, hydroxychloroquine, and azithromycin ST‐T abnormalities including ST elevation were recorded as non‐drug‐induced ECG changes

*Note:* Literature reviews were identified within the literature search and included in the discussion of results.

### 3.3 Results of Individual Studies

**FIGURE 2:**
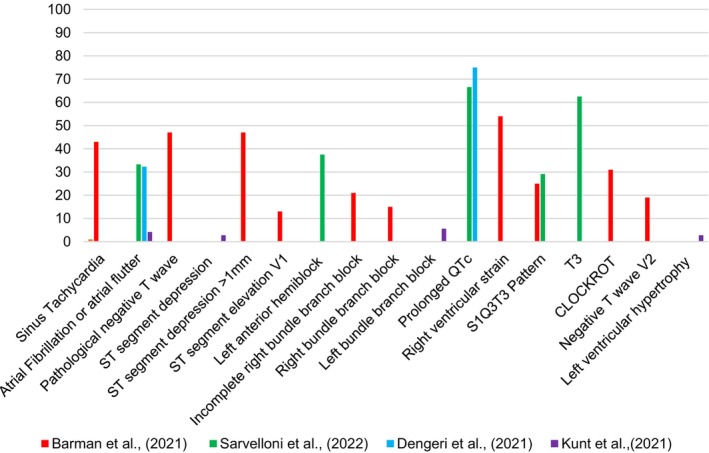
Bar chart displaying the prevalence of statistically significant ECG changes among non‐survivors per study displayed as a percentage value.

Full results for the prevalence of ECG changes between the survivor and non‐survivor groups for each study were tabulated (see Table S3) and the prevalence of ECG changes between the two groups was compared. Statistically significant (*p* < 0.05) ECG changes affecting both survivor and the non‐survivor groups for each included study are available in Appendix S1; Table S5 which was used to formulate Table S6 (presenting the same information as a percentage value). Percentage prevalence for ECG changes among non‐survivors is available in Table S7 which was used to formulate a bar chart to present results (Figure 2).

**FIGURE 3:**
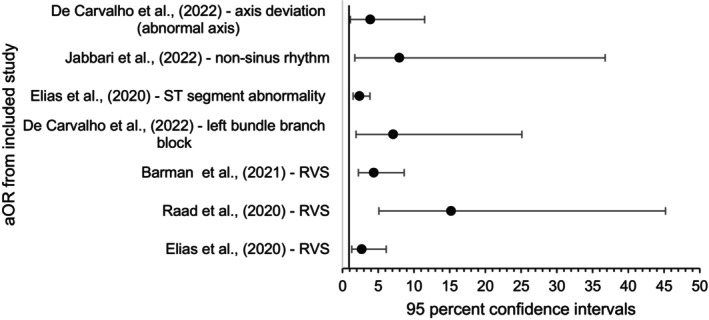
Forest plot displaying statistically significant aORs with 95% CIs (on the *x*‐axis) for the association between ED ECG findings and mortality.

Full results of aORs and aHRs is presented in tabulated format in Appendix S1; Table S9. The Forest plot (see Figure 3) (formulated using data available within Appendix S1; Table S3) shows adjusted ORs for ECG changes with a statistically significant (*p* < 0.05) association with mortality. Savelloni et al. (2022) produced three statistically significant adjusted hazard ratios (HRs) that were not included in the Forest plot to enhance the clinical homogeneity of results. However, these results will be discussed in the synthesis section and are available in a separate Forest plot showing adjusted odds ratios (aORs) and adjusted hazard ratios (aHRs) related to RVS in the data synthesis spreadsheet.

### 3.4 Results of Syntheses

#### 3.4.1 Pooled Analysis

From the eight included studies a total of 3220 patients were included within this SLR, the mean age was 62 years old, and 43.2% of patients were female. Of the 3220 patients, 1802 (87%) survived (survivor group) and 418 (13%) died (non‐survivor group). Further, pooled analysis of results within quantitative synthesis was not performed due to a lack of benefit attributable to the methodological and clinical heterogeneity of the included studies identified within the RoB assessment. Missing data also complicate the synthesis of results. Assumptions made about missing data were difficult due to variations in which ECG rhythms the studies included. For example, missing data relating to sinus rhythm prevalence among survivors compared with non‐survivors was identified within Barman et al. (2021) and Kunt, Kozaci, and Torun (2021) which reduced the combinability of results. It would be difficult and inaccurate to make assumptions about the prevalence of this sinus rhythm within groups. Missing data also made pooled estimates unlikely to add value to this SLR. Due to the different objectives of included studies, there was wide variation in the types of ECG rhythms patients were screened for, with Raad et al. (2021) and Barman et al. (2021) specifically assessing for ECG findings associated with RVS, meaning that data for other ECG findings were not available within their results. This is appropriate for their study methodology due to their objectives, therefore reporting bias is unlikely, but did make pooled analysis for the purpose of this SLR unlikely to be accurate or complete.

A wide range of ECG abnormalities did not produce statistically significant associations with mortality or increased prevalence in non‐survivors. 51 ECG findings were included across the eight included studies so discussing all statistically insignificant findings individually would add little benefit to this SLR. Only statistically significant ECG changes will be reported within this synthesis, but full tabulated results are available in Appendix S1; Table S4.

#### 3.4.2 Right Ventricular Strain (RVS)

Right Ventricular Strain has a statistically significant association with mortality based on aORs produced by three of the included studies (see Appendix S1; Table S2 and Figure S1). Figure 3 shows a variance in certainty of results with Barman et al. (2021) and Elias et al. (2020) producing narrow 95% CIs increasing confidence in results. However, Raad et al. (2021) produced an OR with wide 95% CIs which reduces certainty of the precision of results and the extent to which results may be representative of the population outside of this study. Savelloni et al. (2022) produced an aHR (see Table S2 and Figure S1) (2.95 (CI 1.01–8.55) *p* = 0.047) for the association between RVS on ED admission ECG with 28‐day mortality which supports the findings of Raad et al. (2021), Barman et al. (2021) and Elias et al. (2020).

Individual ECG patterns associated with RVS including S1Q3T3, Q3, CLOCKROT, negative T wave in V2, right bundle branch block (RBBB), and incomplete RBBB were found to have statistically significantly increased prevalence among non‐survivors within results produced by Barman et al. (2021) (see Figure 2).

#### 3.4.3 Atrial Fibrillation (AF)

Savelloni et al. (2022), Kunt, Kozaci, and Torun (2021) and Denegri et al. (2021) found a statistically significant increased prevalence of AF or atrial Flutter on ED ECG among non‐survivors (see Figure 2). Elias et al. (2020) produced a statistically significant aHR for AF (aHR 3.02, CI 1.03–8.81, *p* = 0.042).

#### 3.4.4 Prolonged QTc

Savelloni et al. (2022) and Denegri et al. (2021) reported an increased prevalence of QT prolongation among non‐survivors. Elias et al. (2020) found an association between prolonged QTc interval > 451 ms (aHR 3.24, CI 1.09–9.62, *p* = 0.033) on ED admission ECG and 28‐day mortality.

#### 3.4.5 Abnormal Axis (Axis Deviation)

De Carvelho et al. (2021) found a statistically significant association between abnormal axis and mortality (aOR 3.9, CI 1.1–11.5, *p* = 0.02). This was supported by Jabbari et al. (2023) who found an increased prevalence of left axis deviation among non‐survivors compared with survivors (28.1% vs. 16.5%), unfortunately, they did not report *p* values; therefore, the statistical significance of this finding is unclear. It is also unclear why Jabbari et al. (2023) calculated *p* values for some ECG changes but not others leading to incomplete data and possible reporting bias.

#### 3.4.6 Sinus Rhythm

Denegri et al. (2021) did not produce any aORs or aHRs for predictors of mortality however they did find on multivariate analysis that sinus rhythm at ED admission is a statistically significant independent predictor of increased survival (adjusted HR 2.7, 95% CI 1.1–7.0, *p* = 0.010). This finding was supported by De Carvelho et al. (2021) who found sinus rhythm was associated with an aOR of 0.4 (95% CI 0.2–1.2) but with a statistically insignificant *p* value of 0.08. Jabbari et al. (2023) also supported this finding with a non‐sinus rhythm on ED admission ECG associated with an aOR for mortality of 7.951 when compared with sinus rhythm with a statistically significant *p* value of 0.008 but limited by 95% CIs of 1.924–36.759 highlighting imprecision and decreasing certainty in results.

**FIGURE 4:**
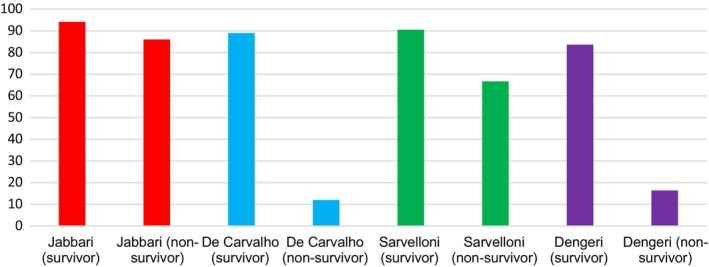
Percentage prevalence of Sinus rhythm within Survivors compared with non‐survivors for studies finding a statistically significant difference between groups.

Four of the included studies showed a statistically significant (*p* < 0.05) increased prevalence of sinus rhythm in survivors compared with non‐survivors (see Appendix S1; Table S8). The statistical heterogeneity evident in Figure 4 may be explained by different definitions of “sinus rhythm” between studies. Sinus rhythm describes any cardiac rhythm originating at the sinus node which can coexist with ECG abnormalities (Garcia and Holtz 2001). This may lead some ECG interpreters to describe an ECG as sinus rhythm whilst categorizing the same ECG to have abnormal findings such as ST‐segment abnormalities. Whereas other studies may have defined “sinus rhythm” as a “normal” ECG with no abnormal findings.

#### 3.4.7 ST Segment and T Wave Findings

Elias et al. (2020) reported a statistically significant association between ST‐segment abnormality and mortality (aOR 2.38, CI 1.49–3.81, *p* < 0.001). Barman et al. (2021) found that ST depression > 1 mm was more prevalent in non‐survivors compared with survivors (47% vs. 24%) as was the presence of a pathologic negative T wave (47% vs. 28%). Kunt, Kozaci, and Torun (2021) also found a statistically significant increased prevalence of ST depression among non‐survivors compared with survivors (2.8% vs. 0.2%) however it is not clear what their criteria for defining ST depression were which reduced the applicability of results to practice.

#### 3.4.8 Left Bundle Branch Block (LBBB)

De Carvalho et al. (2021) found a statistically significant association between LBBB and mortality (aOR 71, CI 1.9–25.1, *p* = 0.002). No other studies assessed for aOR or aHR for LBBB. Kunt, Kozaci, and Torun (2021) found a statistically significant increased prevalence of LBBB among non‐survivors compared with survivors (5.6% vs. 0.9%) however four other studies did not find a statistically significant difference (see Appendix S1; Table S4).

#### 3.4.9 Left Ventricular Hypertrophy (LVH)

Kunt, Kozaci, and Torun (2021) found a small but statistically significant increased prevalence of LVH among non‐survivors compared with survivors (2.8% vs. 1.2%). Two other studies (De Carvelho et al. 2022; Jabbari et al. 2023) investigated LVH prevalence between groups but did not produce *p* values. However, their findings do not imply a difference between groups with De Carvelho et al. (2022) finding a prevalence of 7.1% of LVH in survivors compared with 7% in non‐survivors and Jabbari et al. (2023) finding three cases of LVH on ECG in survivors (1%) and no LVH in non‐survivors.

### 3.5 Risk of Bias (RoB)

#### 3.5.1 Selection Bias

Unavoidable selection bias is present in all included studies due to patients being excluded if they did not have an ECG performed in the ED. More severely unwell or comorbid patients may be likely to have an ECG which could lead to higher mortality rates across all included cohorts than is representative of the true population of COVID‐19 patients that are likely to be assessed in the pre‐hospital environment, which decreases external validity for results of the objectives of this review.

#### 3.5.2 Exposure Measurement

Risk of Bias was identified within the exposure measures relating to the methodology to diagnose COVID‐19. Six of the included studies used reverse transcription polymerase chain reaction (RT‐PCR) to diagnose COVID‐19 whereas Denegri et al. (2021) reported using positive nasopharyngeal swabs to diagnose COVID‐19 however it was not clear which laboratory test was subsequently used. Savelloni et al. (2022) used rapid antigen tests or molecular real‐time PCRs to diagnose COVID‐19. These differences in diagnosis may have led to patients being excluded due to false‐negative results, the prevalence of which varies between diagnostic and laboratory tests for COVID‐19 (Böger et al. 2021).

Interobserver reliability was identified within RoB assessment with the potential for ECG interpretation to vary between clinicians due to their clinical specialty, experience, and expertise. Some studies used emergency physicians to interpret ECGs whereas others used cardiologists or cardiac electrophysiologists. These specialists are likely to be more comprehensive in their interpretation and able to identify subtle ECG abnormalities compared with most pre‐hospital practitioners, which may infer a lack of external validity of the results of this review to pre‐hospital practitioners. Furthermore, not all studies blinded ECG interpreters to data or outcomes which may introduce observer bias.

#### 3.5.3 Confounding Variables

Confounding variables were considered across all studies; however, decisions relating to which comorbidities were included within adjustment within studies that performed multivariate analysis varied. The implications of this are complex and difficult to interpret as COVID‐19 is associated with a considerable number of risk factors for mortality (Dessie and Zawotir 2021). The impact of these risk factors is likely to vary depending on patient characteristics such as frailty, age, and comorbidities (Bhaskaran et al. 2021). Inclusion of every confounding variable would be extremely challenging and interpretation of the clinical significance of these is also difficult. Identification of confounding variables based on retrospective data may also have limitations due to missing data within medical documentation or potential undiagnosed comorbidities among patients. The results of all studies may therefore be affected to an unknown degree by uncontrolled confounding variables which could impact the accuracy of independent association of ECG changes and mortality.

#### 3.5.4 Clinical Heterogeneity

The binary outcome of survival versus non‐survival across all included studies made the accuracy of outcome measurement straightforward; however, time points of outcome measurement varied. Results may therefore be impacted by clinical heterogeneity of the studies due to differences in outcome measures. The majority included studies that used “in‐hospital mortality” as the primary outcome measure, whereas Raad et al. (2021) used mortality, the need for mechanical ventilation, and their composite as the primary outcome measure. To mitigate heterogeneity, only the aOR within their study specifically relating to mortality as an independent outcome was included. This decreases heterogeneity for the purposes of this review but leads to a limitation of unknown association between ECG changes in COVID‐19 patients and morbidity. Savelloni et al. (2022) and Denegri et al. (2021) used mortality at 28 days and 30 days, respectively, as primary outcome measures whereas other studies used in‐hospital mortality as a primary outcome measure with unclear, open‐ended time limits for outcome measurement. These differences may introduce clinical heterogeneity to this review and patients within the survivor groups of studies using 28‐ or 30‐day mortality as an outcome measure may have later died in hospital.

#### 3.5.5 Statistical Heterogeneity

Results of studies varied due to clinical heterogeneity attributable to included studies analyzing different ECG rhythms. Assessment of statistical heterogeneity is therefore limited and challenging. Eyeball test of the Forest plot (Figure 3) shows that the CIs for the three aORs relating to RVS and mortality overlap indicating a degree of homogeneity. However, other aORs related to different individual ECG rhythms therefore providing no directly comparable results. Analysis of statistical heterogeneity among results relating to prevalence as a percentage was again limited by clinical heterogeneity of included studies screening for different ECG rhythms and the presence of missing data. Due to the clinical heterogeneity of included studies combined with a small number of studies producing comparable results, meta‐analysis and statistical analysis of heterogeneity is unlikely to be accurate or useful.

#### 3.5.6 Reporting Biases

Missing data was identified within the aORs produced by De Carvalho et al. (2021); however, this can be explained as the authors chose to only complete multivariate regression analysis on variables with a *p* value < 0.10 within the univariate analysis. Therefore, the missing aORs would not have been statistically significant. Reporting bias was considered; however, due to the authors' rationale, deemed to be of low risk.

For Barman et al. (2021) the objective of their study was to assess the relationship between RVS on mortality in COVID‐19 patients so although the omission of sinus rhythm from their review causes missing data within this review it does not imply reporting bias because they chose to include only ECG findings associated with RVS. Kunt, Kozaci, and Torun (2021) omitted to report the prevalence of sinus rhythm among survivors compared with non‐survivors and the reason for this is less clear. This missing data can potentially be explained as their review focused on a broader range of mortality predictors than ECG findings alone as they also reported laboratory and computerized tomography findings so performed a less detailed analysis of ECG findings compared with other studies that focused exclusively on ECG findings.

### 3.6 Certainty of Evidence

Certainty was assessed throughout based on CIs which are presented within synthesis and tables where available. As evident in the Forest plot (Figure 3), the CIs varied widely between ORs. There appears to be an association between some ECG abnormalities and mortality in COVID‐19 patients with CIs overlapping, especially in the RVS pattern where three studies found a statistically significant aOR associated with mortality with CIs overlapping; however, the precision of these calculated ORs is less certain due to wide CIs.

### 3.7 Ethics

All included studies excluding Denegri et al. (2021) gained ethical approval from an ethics committee. Due to the noninterventional and retrospective methodology used by all studies and no identifiable patient information included, there were no significant ethical problems identified. The only included prospective study by Kunt, Kozaci, and Torun (2021) gained informed consent from participants.

## 4 Discussion

ECG abnormalities in COVID‐19 patients are likely to be due to cardiovascular involvement and associated myocardial injury or underlying cardiovascular disease (Liu et al. 2023). Development of arrhythmias has been observed in hospitalized COVID‐19 patients and is associated with increased disease severity and higher mortality, especially in patients with new‐onset AF or atrial flutter (The Task Force for the Management of COVID‐19 of the European Society of Cardiology 2022). Furthermore, patients with pre‐existing AF are at a higher risk of mortality from COVID‐19 (Zuin et al. 2021). The results of this SLR support that AF was more prevalent in COVID‐19 non‐survivors and has an independent association with mortality based on an aOR found in one included study. The exact mechanisms by which COVID‐19 causes AF are not fully understood and are likely to occur through multiple pathophysiological mechanisms including inflammatory signaling causing cytokine storm, reduction in angiotensin‐converting enzyme 2 receptor availability, CD147‐ and sialic acid‐spike protein interaction, endothelial damage, electrolyte or acid–base abnormalities, and increased adrenergic drive (Chen et al. 2022; Gawałko et al. 2020). AF may subsequently increase mortality through the development of a prothrombotic state (Gawałko et al. 2020).

A narrative literature review by Angeli et al. (2022) identified a high prevalence of venous thromboembolism reported among hospitalized COVID‐19 patients. This is secondary to a hypercoagulable state that has been observed in COVID‐19 patients and increases the risk of pulmonary embolism (PE) (Tang et al. 2020). PE is estimated to affect 2.6%–8.9% of hospitalized COVID‐19 patients increasing to a third in those admitted to the intensive care unit (Sakr et al. 2020). ECG findings such as sinus tachycardia and changes associated with acute right‐sided heart failure, pulmonary hypertension, and RVS are seen in COVID‐19 patients which may indicate associated PE (Chen et al. 2022). However, non‐specific ST‐segment and T‐wave changes are more common among COVID‐19 patients with PE (Angeli et al. 2022). This supports the findings of this SLR and may explain why some of the included studies found an increased mortality associated with RVS and ST‐segment abnormalities. It is less clear how the findings from these reviews, including hospitalized patients, translate to pre‐hospital practice; however, a Scottish nationwide self‐controlled case‐series study carried out by Ho et al. (2021) assessed 30,709 with confirmed COVID‐19 in hospitals and the community for association with thrombotic events. Ho et al. (2021) found an increased incidence of thromboembolisms including PE, deep vein thrombosis, myocardial infarction, and ischemic strokes in both hospitalized patients and patients in the community which lasts for at least 8 weeks after infection. Patients not originally hospitalized for COVID‐19 had an incidence rate ratio for thromboembolism of 4.07 (95% CI, 2.83 to 5.85) (Ho et al. 2021). This is a concerning finding for pre‐hospital healthcare providers because the symptoms of COVID‐19 and PE can sometimes be difficult to differentiate, especially in severe cases (Hesam‐Shariati et al. 2021). Therefore, assessing ECGs for changes associated with PE is advisable for patients assessed in the pre‐hospital environment and could help to detect COVID‐19 patients requiring treatment for PE.

Repolarization abnormalities such as increased QRS and QTc intervals have been identified as early markers for COVID‐19 disease progression and mortality in hospitalized patients (Wawrzeńczyk and Grabowski 2022). QTc prolongation is especially concerning as it is associated with an increased risk of ventricular arrhythmias (Thakore et al. 2021). The findings of this SLR support this with prolonged QTc and LBBB found to be increasingly prevalent among non‐survivors and independently associated with mortality. QTc prolongation in COVID‐19 may be medication induced or due to increased cytokine which can lead to stimulation of myocyte ion channels and inhabitation of enzyme CYP3A4 or due to abnormal electrolytes including hypokalemia or hypomagnesemia (Pornwattanakavee, Priksri, and Leelakanok 2021). Jabbari et al. (2023) did not include data regarding the cause of death among the included population, they concluded that patients presenting with non‐sinus rhythm at ED are at an increased mortality and the authors suggested that these patients should have continuous ECG monitoring since this might provide important prognostic information. A nationwide self‐controlled case series and matched cohort study conducted in Sweden by Katsoularis et al. (2023) included 1,057,174 exposed (COVID‐19) individuals and 4,074,844 matched unexposed individuals. The authors found that the exposed population was at a significantly increased risk of cardiac arrhythmias including atrial tachycardias, paroxysmal supraventricular tachycardias, and bradyarrhythmia following COVID‐19 infection. However, they did not find an increased risk of ventricular arrhythmias following COVID‐19 infection, this could be explained by methodological limitations including that the sample size for this outcome was insufficient due to “day zero” events being excluded from the analysis, as were individuals with pre‐existing cardiovascular diagnosis of the same form. Day zero events were defined as arrhythmias occurring on the same date as the COVID‐19 date (day zero). The authors excluded these events due to the high number of day zero events because they introduce bias to the results and may be associated with reverse causation such as hospital‐acquired COVID‐19 infection whilst the patient is hospitalized for an arrhythmia event. Therefore, despite the large sample size, it is difficult to interpret the findings of this study in the context of ventricular arrhythmias and COVID‐19.

Kunt, Kozaci, and Torun (2021) did not report the cause of death or prevalence of ventricular arrhythmia among the included population, however, they did report longer QTc times in patients in the non‐survivor group. There may be an increased risk of ventricular arrhythmia in these patients which might explain the increased mortality rate, although due to missing data, the prevalence of this is unclear. Patients with long QT syndrome are more at risk of arrhythmias when infected with COVID‐19 thus they should receive cardiac monitoring due to the high risk of QT prolongation seen in COVID‐19 patients (Dherange et al. 2020). COVID‐19 itself is independently associated with a significant increase in QTc and a QTc > 500 ms, irrespective of the use of treatment with medications associated with QTc prolongation (Lavelle, Desai, and Wan 2022). This may be due to viral channelopathies whereby viral infection causes ion channel dysfunction which may result in long QT‐related arrhythmias (Lavelle, Desai, and Wan 2022); however, a review by Dherange et al. (2020) discussed the causes of arrhythmias including ventricular arrhythmias could be due to a number of mechanisms including; hypoxia caused by direct tissue involvement of lungs, myocarditis, abnormal host immune response, myocardial ischemia, myocardial strain, electrolyte derangements, intravascular volume imbalances, and drug sides effects. Thus, although further research is required to understand the relationship between COVID‐19 and ventricular arrhythmias, care should be nuanced to the patient presentation with the cause of their arrhythmia considered and treated appropriately. Furthermore, patients presenting with prolonged QTc or showing signs of myocardial strain or ischemia may be at an increased risk of ventricular arrhythmias and may benefit from continuous cardiac monitoring.

The results suggest sinus rhythm is associated with decreased mortality and COVID‐19. These findings may have limited applicability to practice due to no consistent or clear definition of sinus rhythm between included studies. Sinus rhythm can coexist with other ECG abnormalities, so it is not clear whether the presence of sinus rhythm is associated with decreased risk if other ECG abnormalities are present. Although results imply that sinus rhythm is more prevalent among survivors in the included studies it would be inappropriate to conclude that the presence of sinus rhythm is sufficient to consider a patient low risk due to uncertainty and clinical heterogeneity between included studies.

ST‐segment and T‐wave changes were found within this SLR to be more prevalent among non‐survivors compared with survivors. ST‐segment changes were the most frequently reported ECG finding in a literature review and meta‐analysis of ECG changes occurring during hospitalization among COVID‐19 patients by Garcia‐Zamora et al. (2021) who highlighted that these ECG findings are associated with multiple pathologies including pericarditis, acute coronary syndrome and Takotsubo cardiomyopathy. It appears appropriate to recommend that COVID‐19 patients assessed in the pre‐hospital environment should receive an ECG assessment to evaluate for these findings because these conditions may require in‐hospital assessment and treatment.

The causality of cardiac complications in COVID‐19 patients is likely to be multifactorial and dependent on factors including pre‐existing comorbidities such as underlying cardiovascular disease, age, individual predisposition, immune response, inflammatory burden, and systemic pathological conditions (Nascimento and Sable 2022). Whilst causality may be unclear, the association between some ECG abnormalities and mortality appears to be statistically significant. Because there is a known association between cardiovascular involvement and mortality, the ECG provides a non‐invasive, easy and accessible tool to assist in identifying high risk patients (Chen et al. 2022). It may not be immediately clear whether the ECG changes are acute or due to a patient's comorbidities (Duca et al. 2022); therefore, ECGs should be interpreted as part of a thorough patient assessment and history taking.

### 4.1 Limitations of Evidence

#### 4.1.1 Confounding Variables

The comparison of the prevalence of ECG changes between groups within studies provides information about association; however, causality cannot be inferred from these results. Furthermore, the prevalence of ECG changes between the two groups is likely to be impacted by confounding variables although the clinical significance of this is unclear. The reason for differences in the prevalence of these ECG changes between survivors and non‐survivors is likely to be multifactorial due to a range of covariates including comorbidities, severity of COVID‐19, and related complications. Despite these limitations, the ECG is likely to be a useful clinical assessment tool in COVID‐19 patients which adds information regarding the patient's condition and enhances prognostication, but it should be interpreted alongside thorough patient assessment, history taking, and clinical judgment in the context of the patient's presentation and medical history.

None of the studies included within this literature review reported the prevalence of pulmonary embolism (PE) among the included populations despite PE being one of the most commonly occurring thrombotic complications affecting COVID‐19 patients (Zhan et al. 2022) and is therefore likely to be a significant confounding variable. COVID‐19 is a multiorgan disease associated with both short‐term and long‐term complications including myocardial infarction, stroke, venous thromboembolism, and bleeding (Katsoularis et al. 2022). Thus, it would be beneficial to understand the association between COVID‐19, PE, and ECG abnormalities such as RV strain, RBBB, and S1Q3T3 which may be associated with PE. The cardiovascular complications described by Katsoularis et al. (2022) are likely to be associated with several ECG abnormalities, and the clinical significance of these and their association with mortality and cause of death requires further research to aid understanding and interpretation of results. Savelloni et al. (2022) did not report the prevalence of PE in the included population; however, they reported that S1Q3T3 or inverted T wave on ECG recordings was independently associated with 28‐day mortality. Furthermore, the T3 sign alone was included in the right ventricular strain analysis as a strain mark and was found to be associated with poor outcomes. The presence of PE could explain these findings; however, other pulmonary pathologies may be associated with these findings. Barman et al. (2021) did not report the prevalence of PE in the included population although they discussed that PE could explain the association between RVS ECG changes and mortality although other pathophysiological mechanisms may also explain this association including that COVID‐19 infection can cause RV overload due to both lung and systemic inflammation. Barman et al. (2021) also highlighted that a limitation of using RV strain pattern as a prognostic tool in COVID‐19 patients is that it can occur in approximately 10% of the normal population.

Raad et al. (2021) found that ECG changes suggestive of right heart strain (RHS) were associated with worse outcomes including mortality and they did not report the cause of death of these patients; however, the authors discussed that PE may explain these findings. However, other causes of RV dysfunction such as respiratory failure, hypoxic vasoconstriction, or physical destruction of the capillary beds that lead to elevated pulmonary vascular resistance may also explain the association between RHS ECGs and mortality (Raad et al. 2021). Raad et al. (2021) reported that acute pulmonary emboli were not well accounted for within their research because only 2 patients received a dedicated computed tomographic pulmonary angiography. They concluded that RVS ECGs were associated with worse outcomes in COVID‐19 patients; however, the etiologies of this could not be investigated within their research.

PE could be a cause of ECG changes associated with RVS; however, other pulmonary pathologies including respiratory failure and pulmonary hypertension may also cause these findings. Larger prospective studies are likely to be required to further understand the prognostic significance of RV strain, S1Q3T3, and RBBB on initial ECG in COVID‐19 patients and the cause of these findings in COVID‐19 patients. Thus, a limitation of this literature review is that although the findings suggest a correlation, causation cannot be determined from the results.

#### 4.1.2 Baseline ECG Abnormalities

Unfortunately, none of the included studies compared the ECG at ED presentation with the patient's baseline ECG. Baseline ECG abnormalities may reflect comorbidities which may increase the risk of mortality. Therefore, whether mortality is associated with preexisting comorbidities or as a direct result of the COVID‐19 illness or associated complications cannot be extrapolated from this literature review. Further research comparing baseline ECGs and new ECG abnormalities would be required to determine whether a change in ECG compared to baseline reflects causes associated with the current illness. This research would likely be very difficult to achieve as many patients will not have previous ECGs available, and where they are available it would be difficult to determine whether these changes have occurred since the previous ECG or during the time they have been infected with COVID‐19.

#### 4.1.3 Exclusion of Patients With No ECG Performed in the ED

Studies excluded patients who did not have an ECG performed in ED which introduces selection bias because more severe patients may be likely to have an ECG recorded. From a pre‐hospital perspective, ECGs would not routinely be undertaken when assessing COVID‐19 patients; however, patients presenting with dyspnea or chest pain would be more likely to receive an ECG. The correlation of symptoms such as dyspnea and chest pain as prognostic indicators for COVID‐19 patients has not been assessed within this review and may be a confounding variable within the studies as ECGs on included patients may have been undertaken in the ED due to the presence of these symptoms.

#### 4.1.4 External Validity

The 8 included studies were undertaken across 6 different countries, thus there may be variations across populations and healthcare systems between these studies which may reduce the generalizability and external validity of results across different healthcare settings. There may be differences in baseline characteristics of populations such as prevalence of comorbidities and due to changes in COVID‐19 disease throughout the pandemic such as COVID‐19 strain and severity of illness. Furthermore, differences in vaccination rates between populations may influence results with variation between countries regarding access to vaccinations, especially as all included studies obtained their data within 2020 or 2021. Therefore, it is unclear how representative this data obtained within the height of the pandemic will be of patients now thus results may lack generalizability to current COVID‐19 patients. Furthermore, none of the studies included within the review included ethnicity as a risk factor for mortality despite evidence showing that people from non‐white ethnic groups are at a higher risk for mortality (Bhaskaran et al. 2021). It is therefore unclear based on the evidence included within this review whether ethnicity is a risk factor for ECG abnormalities in COVID‐19 patients.

#### 4.1.5 Observational Studies as a Methodology

Observational studies provide less reliable evidence compared with randomized controlled trials due to confounding variables and bias (Hackshaw 2015). Seven of the included studies used retrospective methodology which further decreases the reliability of results (Curtis and Drennan 2013). Whilst these limitations of the evidence must be acknowledged, this appears unavoidable due to being the most appropriate methodology for the objectives of the studies. However, there were controllable methodological limitations including that only three of the included studies reported blinding ECG interpreters to outcomes. The remaining studies could have improved validity by using blinding of the ECG interpreters to the outcome data to prevent observer bias.

### 4.2 Limitations of the Review Process

The review was limited by the use of a single researcher and lack of meta‐analysis which was not performed due to the complexity of data, the presence of missing data, and the lack of clinical heterogeneity.

This review is limited by indirectness due to the use of data from EDs due to a lack of pre‐hospital evidence. This may mean that the included populations are not entirely representative of the patients assessed in the pre‐hospital environment. It is not clear within the included studies how many patients in the ED made their own way to the hospital and how many were brought in by ambulance, this may cause variances in the population baselines and disease severity with pre‐hospital practitioners being potentially more likely to assess a frailer, more comorbid, or severely unwell population who cannot make their own way to hospital.

### 4.3 Research Implications

While some studies produced statistically significant differences in prevalence in survivors compared with non‐survivors and statistically significant aORs and aHRs showing an independent association between some ECG changes and mortality, these findings only highlight an association; causality cannot be inferred from the results. The transferability of this to clinical practice may be difficult due to unclear causation, for example, ECG changes may be the result of comorbidities and it may be unclear whether the patient had a previously undiagnosed cardiac or respiratory history which may have caused the ECG changes or whether they are related to COVID‐19 or complications of COVID‐19.

The findings of this SLR support ECG assessment of these patients to screen for findings associated with increased mortality including non‐sinus rhythm, QTc prolongation, and other repolarization abnormalities including LBBB, axis deviation, AF or atrial flutter, RVS patterns, ST segment changes and T‐wave abnormalities, and evidence of LVH. Conversely, sinus rhythm is associated with decreased mortality. ECG assessment therefore provides some prognostic value in the assessment of these patients. Although as a standalone assessment ECGs may not change decision‐making, they could help form a clearer understanding of the patient's condition and risk factors which can aid clinical judgment. The research supports the assessment of ECGs in patients presenting with COVID‐19 and appears to have some value as a tool for prognostication.

However, using the RVS pattern as a prognostication tool in COVID‐19 patients may have limitations due to RVS patterns such as complete and incomplete RBBB being present in 10% of the normal population (Barman et al. 2021). Thus, access to previous ECGs for patients may be necessary to assess whether the change is acute or known. Some patients have ECGs available in the pre‐hospital environment through previous patient care records; however, many will not have this information available.

## 5 Conclusion

This review identified studies of varying quality and with RoB and heterogeneity identified, therefore further research is required to better understand the association of pre‐hospital ECG changes in COVID‐19 patients and mortality. ECG abnormalities identified on pre‐hospitalization ECG are associated with increased mortality in COVID‐19 patients. These ECG abnormalities include non‐sinus rhythm, QTc prolongation, left bundle branch block, axis deviation, atrial fibrillation, atrial flutter, right ventricular strain patterns, ST segment changes, T‐wave abnormalities, and evidence of left ventricular hypertrophy. Although the results of this review have limitations that make interpretation of the results difficult, pragmatically ECGs are quick, cheap, non‐invasive, readily available, and may be beneficial when assessing COVID‐19 patients in the pre‐hospital environment by assisting in the identification of patients at increased risk of mortality. The interpretation of these abnormalities needs to be nuanced to the patient and their presentation as abnormalities could be due to patient comorbidities, past medical history, or acute pathology including cardiovascular complications or secondary thrombotic events such as PE. Therefore, ECGs may help guide decision‐making and assessment, but due to the broad range of patients assessed and the range of severity of illness seen in COVID‐19 patients, no single diagnostic test or triage tool can be relied on independently to stratify risk. Patients with ECG changes associated with ventricular arrhythmia such as prolonged QTc may benefit from continuous cardiac monitoring. Patients should receive thorough history taking, and physical assessment alongside the use of clinical triage tools and clinical judgment to prognosticate and make clinical decisions alongside the patient's wishes and based on their best interests.

## Author Contributions

Danielle Askey did this project as a MSc programme and did all the analysis. Ann Smith was her supervisor and proof read the article.

## Ethics Statement

Ethical approval not required as systematic literature review methodology used with no primary research undertaken.

## Conflicts of Interest

The authors declare no conflicts of interest.

## Supporting information


Appendix S1.

## Data Availability

Supporting Information submitted will provide full collected data.
