# Supplementary material for: Are Pre‐Hospitalization ECG Abnormalities Associated With Increased Mortality in COVID‐19 Patients? A Quantitative Systematic Literature Review
Source: Ann Noninvasive Electrocardiol. 2024 Oct 12;29(6):e70016. doi: 10.1111/anec.70016 (PMC11470194; doi:10.1111/anec.70016)
Supplement: Supplementary file 1 — Appendix S1. [file ANEC-29-e70016-s001.zip › anec70016-sup-0001-Tables.docx]

Supplementary information 1. Table S1. CASP Checklist Risk of Bias Assessment

|  | **Included Study** | | | | | | | |
| --- | --- | --- | --- | --- | --- | --- | --- | --- |
| **CASP Checklist** | **Elias *et al.,* (2020)** | **Jabbari *et al.,* (2022)** | **De Carvalho *et al.,* (2022)** | **Raad *et al.,* (2020)** | **Barman *et al.,* (2021)** | **Savelloni *et al.,* (2022)** | **Denegri *et al.,* (2021)** | **Kunt *et al.,* (2021)** |
| **Did the study address a clearly focused issue?** | Y | Y | Y | Y | Y | Y | Y | Y |
| **Was the cohort recruited in an acceptable way?** | Y = retrospective | Y = retrospective | Y = retrospective | Y = retrospective | Y = retrospective | Y = retrospective | Y = retrospective | Y = prospective |
| **Was the exposure accurately measured to minimise bias?** | N - Reverse transcription polymerase chain reaction (RT-PCR) to diagnose COVID-19. ECGs interpreted by electrophysiologists, no evidence that blinding was used. | N = RT-PCR. Unclear who interpreted ECGs, no evidence that blinding was used. | N = RT-PCR. ECGs interpreted by 2 independent emergency physicians (2 hospitals) and an emergency physician and cardiologist (1 hospital). No evidence that blinding was used for ECG interpreters. | Y = RT-PCR used. 2 medical doctors (1 working within a cardiology department and another in a cardiac electrophysiology department) also authors of the study) interpreted ECGs and were blinded to data and outcomes. | Y = RT-PCR used, 2 independent cardiologists interpreted ECGs and were blinded to clinical data and study design. Interobserver concordance rate was 98% | Y = rapid antigen test or molecular real time PCR. ECGs interpreted by a specialised cardiologist who was blinded to outcomes. | N = positive nasopharyngeal swab, unclear which laboratory test used. Unclear who interpreted ECGs and no evidence of blinding of ECG interpreters. | N = RT-PCR. ECGs interpreted by an emergency medicine instructor; no evidence of blinding used. |
| **Was the outcome accurately measured to minimise bias?** | Y = the binary outcome of survival vs non-survival across all included studies made accuracy of outcome measurement simple. However, time points of measurement varied slightly between studies (see Table 1.). | Y | Y | Y | Y | Y | Y | Y |
| **Have the authors identified all important confounding factors?** | Y = demographic, comorbidities, vital signs were identified as confounding variables. | Y = demographics (age and sex), current and former smoking status, underlying diseases (IHD, VHD, cardiomyopathy, asthma, COPD, CVA, malignancy and ESRD. | Y - demographics (age, sex), comorbidities (diabetes, CAD, HF, arrhythmia, tobacco use, COPD, CKD, stroke, HTN, anticoagulant and antiplatelet therapy, antiarrhythmic therapy, and vital signs. | Y = the authors reported including statistically and clinically relevant variables within the multivariate analysis associated with COVID-19 critical illness based on two risk factor analyses undertaken in China including a nationwide analysis. | Y = As the authors were investigating RVS specifically they excluded patients with known cardiomyopathy, pulmonary hypertension, HF, severe valvular disease, previous PE, and severe COPD. Demographic characteristics such as gender, age, smoking, known hyperlipidaemia, HTN, diabetes were identified as confounding variables. | N = Comorbidities were adjusted for however confounding factors such as sex and age do not appear to have been adjusted for within adjusted HRs which may introduce confounding variables. | CT = the authors identified hypertension, diabetes, coronary artery disease (CAD), chronic kidney disease (CKD) and atrial fibrillation as cofounding variables. Other variables may also have an impact on mortality however inclusion of every single co-morbidity which may impact mortality would be difficult to achieve. | N = the authors compared groups for chronic diseases and vital signs as well as ECG changes but only compared prevalence of findings between groups without adjusting for covariates. |
| **Have they taken account of the confounding factors in the design and/or analysis** | Y - ORs were adjusted at multivariate analysis adjusting for demographics, comorbidities, and vital signs. | CT = ORs were adjusted to account for confounding variables however unclear which. It is likely that they adjusted for the demographics, smoking status and comorbidities identified above. | Y = adjusted ORs were produced following multivariate logistic regression analysis, it is not clear how ORs were adjusted but likely that they used the above confounding variables. | Y = as above the covariates included in the design appear to be thoroughly considered. | Y = the authors used multivariate cox regression analysis to produce and adjusted HR and adjusted OR for RVS on ED ECG and mortality. | Y = HRs produced from multivariate Cox hazard regression model analysis were adjusted for were severity of COVID-19 (expressed as PaO2/FiO2 ratio <300) and comorbidities of CAD, HTN, CHD, CHF. | Y = ORs were adjusted for within multivariate analysis for age, sex, diabetes, CAD, resuscitation, and medical critical area admission. | N = ECG findings were compared between survivors and non-survivors for prevalence but no adjusted ORs or HRs were produced. Instead, they used unadjusted area under curve (AUC) as a measure of sensitivity and specificity for mortality. |
| **Was the follow up of subjects complete enough?** | Y - across all included studies, all patients who met the studies inclusion criteria were accounted for within the analyses. | Y | Y | Y | Y | Y | Y | Y |
| **Was the follow up of subjects long enough?** | N - outcome measurements taken at 48 hours which compared with other studies does not appear to be a long enough period to assess impact of initial ECG on mortality. Some patients in the survivor group may have died beyond 48 hours meaning the non-survivor group was not representative of the true population. | Y = 30-day mortality used as timepoint for outcome measurement. | Y = in-hospital mortality used as outcome measurement with unclear time frame. | Y - no time frame given for measuring of mortality, authors appeared to simply use any in-hospital mortality as the outcome measure. | Y = death during hospitalisation used as outcome measure but unclear over which time point this was measured. | Y = 28-day mortality use as time point for outcome measurement. | Y = 30-day mortality used as time frame for outcome measurement. | CT = prospective but unclear when outcome measurements were taken |
| **What are the results of this study?** | RVS (OR 2.7, 95% CI 1.30-6.12, *P* 0.007) and ST segment abnormalities (OR 2.38, 95% CI 1.49-3.84, *P* <0.001) were statistically significantly associated with mortality. | A non-sinus rhythm on admission ECG is associated with increased 30-day mortality than a sinus rhythm (adjusted OR 7.961, *P* 0.008) | Abnormal axis (adjusted OR 3.9, 95%CI 1.1-11.5) P 0.02) and Left Bundle Branch Block (LBBB) (adjusted OR 7.1, 95% CI 1.9-25.1, *P* 0.002) were statistically significantly associated with mortality. | This study implies that right heart strain (RHS) patterns identified on initial ECG are associated with increased mortality. | RVS on ECG was associated with an OR of 4.385, *P* <0.001 | QTc interval >451ms, RVS, and AF are associated with statistically significant increased mortality in COVID-19 patients. | Sinus rhythm at ED admission ECG is associated with increased 30-day survival. Other ECG abnormalities were associated with mortality at univariate but did not produce statistically significant results at multivariate analysis. | There was a statistically significant (P <0.001) increased prevalence of atrial fibrillation, left bundle branch block, left ventricular hypertrophy and ST segment depression in non-survivors. The AUC for ECG QTc was 0.347 indicating poor sensitivity and specificity. |
| **How precise are the results?** | CI intervals were wide implying imprecision of results. | The above finding produced a wide 95% CI (1.724-36.759) which implies impression of results. | The CIs were wide implying impression of results. | The adjusted OR for RHS ECG and mortality is 11.4 (95% CI, 5.2-24.9). The wide CIs indicate a lack of precision in the results. | The 95% CI for the above result is 2.226-8.638 representing a wide CI and reducing precision of results. | QTc interval >451ms (adjusted HR 3.24, 95% CI 1.09-9.62), RVS (adjusted HR 2.94, 95% CI 1.01-8.55) and AF (adjusted HR 3.02, 95% CI 1.03-8.81) are associated with increased mortality in COVID-19 patients. The CIs across all findings are wide implying imprecision in results. | 95% CI interval 0.02-0.27 for sinus rhythm indicating a narrow CI and precise results. | The AUC for QTc 95% CI was 0.226-0.467 indicating reasonable precision of results however performing poorly on the AUC. |
| **Do you believe the results?** | Yes - right ventricular overload appears to be associated with statistically significant mortality across a number the included studies. | Yes - despite flawed methodology and RoB identified, the conclusion that a non-sinus rhythm is associated with greater mortality than a sinus rhythm is believable as this would imply comorbidities or increased severity of disease in patients with non-sinus rhythm. | Yes - the methodology appears robust although there could be observer bias due to a lack of blinding, axis deviation and LBBB on ECG could imply underlying undiagnosed comorbidities or increased severity of COVID-19 which could increase morbidity. | Yes - the findings are statistically significant and fit with other findings although lack of precision may reduce confidence in results. | Yes - RVS appears to be associated with increased mortality in COVID-19 patients within many of the included studies. | Yes - there is evidence of imprecision of results, but the results fit with other studies and findings of AF, prolonged QTc and RVS are likely to suggest cardiac involvement, comorbidities or increased severity of disease which could increase the risk of mortality. | Yes - the statistically significant findings imply sinus rhythm on initial ECG reduces risk of mortality. | Yes - the results show increased prevalence of ECG abnormalities among non-survivors which is not surprising however the value of these findings as a prognostic tool associated with mortality is limited as no adjusted ORs or HRs were produced so confounding variables influence results. |
| **Can the results be applied to the local population?** | CT - data obtained within 2020 in New York at the height of the COVID-19 pandemic which may therefore not be generalisable across different counties, healthcare systems or current COVID-19 patients. | CT = data was obtained in Iran in 2021, the severity of illness and population demographics, results may therefore not be generalisable across different counties, healthcare systems or current COVID-19 patients.. | CT - data obtained from 3 hospitals in France in 2020, thus results may not be generalisable across different counties, healthcare systems or current COVID-19 patients. | CT = data obtained in 2020 in the USA results may not be generalisable across different counties, healthcare systems or current COVID-19 patients. | CT - data obtained in 2020 in Turkey thus results may therefore not be generalisable across different counties, healthcare systems or current COVID-19 patients. | CT = data was obtained from a cohort in Italy in 2020-2021 implying severity of disease was likely to be increased and vaccination programmes not likely to be initiated, therefore results may not be generalisable across different counties, healthcare systems or current COVID-19 patients. | CT - data obtained from Italy in 2020, thus results may not be generalisable across different counties, healthcare systems or current COVID-19 patients. | CT - data obtained in 2020-2021 in Turkey in an area highly populated by tourists, therefore results may not be generalisable across different counties, healthcare systems or current COVID-19 patients. |
| **Do the results of this study fit with other available evidence?** | Yes - right ventricular overload appears to be associated with statistically significant mortality across a number the included studies. | Y = other included studies support this evidence but with variable statistical significance. | Y = axis deviation may suggest RVS which fits with the findings of other studies. LBBB does not appear to be statistically significantly associated with morbidity in other included studies. | Y = other studies have found an association between RHS (synonymous with RVS) and mortality | Y = other studies within this SLR have also found a statistically significant association between RVS on ED ECGs and mortality in COVID-19 patients. | Y = other included studies also found increased association between QTc prolongation, AF and RVS and mortality in COVID-19 patients. | Y = Jabbari et al., (2022) found that a non-sinus rhythm is statistically significantly associated with increased mortality. | Yes - other studies have shown increased prevalence of ECG abnormalities in non-survivors compared with survivors (see supplementary information 2 Table S5. |
| **What are the implications of this study to practice?** | ECG assessment of COVID-19 patients to assess for RVS and ST segment abnormalities may assist in prognostication of these patients. | ECG assessment of COVID-19 patients may provide useful prognostication with identification of a non-sinus rhythm compared with sinus rhythm being associated with increased mortality. | Early ECGs in COVID-19 patients may be helpful in predicting mortality. Identification of abnormal axis and left bundle branch block was associated with statistically significant mortality in this study. | This study implies that right heart strain patterns identified on initial ECG are associated with increased mortality. | This study supports that ECGs should be undertaken in COVID-19 patients and signs of RVS may be add prognostic value for mortality. | Identification of QTc > 451ms, AF or RVS on initial ECG in COVID-19 patients is associated with increased mortality. ECGs should be assessed for these abnormalities to assist in risk stratification. | Sinus rhythm at ED admission reduces the risk of mortality, implying that non-sinus rhythms may on the contrary be associated with increased mortality. ECG assessment is therefore valuable in prognostication. | ECG abnormalities may be more likely to be prevalent in patients who go on to die however it is unclear how much this is influenced by confounding variables. |
| **RoB Quality of evidence: (high, medium, or low-quality evidence)** | Medium | Low | Medium | High | High | Medium | Medium | Low |

Table S2. Right ventricular strain adjusted odds ratios and adjusted hazard ratio for Savelloni *et al.,* (2022).

|  | aOR | Lower CI | Graph Lower 95% CI | Upper CI | Graph Upper 95% CI | Position* |
| --- | --- | --- | --- | --- | --- | --- |
| Elias - Right Ventricular Strain | 2.7 | 1.3 | 1.4 | 6.12 | 3.42 | 0.5 |
| Raad - Right Ventricular Strain | 15.2 | 5.1 | 10.1 | 45.2 | 30 | 1.5 |
| Barman - Right Ventricular Strain | 4.385 | 2.226 | 2.159 | 8.638 | 4.253 | 2.5 |
| Savelloni (adjusted Hazard Ratio) - Right Ventricular Strain | 2.94 | 1.01 | 1.93 | 8.55 | 5.61 | 3.5 |

*Position as column within tables referring to position of data on Forest Plot to allow graph formulation.

Table S3. Statistically significant adjusted odds ratios and 95% confidence intervals for association between ED ECG findings and mortality.

|  | OR | Lower CI | Graph Lower 95% CI | Upper CI | Graph Upper 95% CI | Position |
| --- | --- | --- | --- | --- | --- | --- |
| Elias et al., (2020) - RVS | 2.7 | 1.3 | 1.4 | 6.12 | 3.42 | 0.5 |
| Raad et al., (2020) - RVS | 15.2 | 5.1 | 10.1 | 45.2 | 30 | 1.5 |
| Barman et al., (2021) - RVS | 4.385 | 2.226 | 2.159 | 8.638 | 4.253 | 2.5 |
| De Carvalho et al., (2022) - left bundle branch block | 7.1 | 1.9 | 5.2 | 25.1 | 18 | 3.5 |
| Elias et al., (2020) - ST segment abnormality | 2.38 | 1.49 | 0.89 | 3.84 | 1.46 | 4.5 |
| Jabbari et al., (2022) - non-sinus rhythm | 7.96 | 1.724 | 6.236 | 36.759 | 28.799 | 5.5 |
| De Carvalho *et al.*, (2022) - axis deviation (abnormal axis) | 3.9 | 1.1 | 2.8 | 11.5 | 7.6 | 6.5 |

Data used for formulation of Figure 3. Forest Plot displaying statistically significant aORs with 95% CIs (on x axis) for association between ED ECG findings and mortality.

Table S4. ECG changes and prevalence in survivors compared with non-survivors (*P* values included where available).

| **ECG finding** | **Study by lead author** | | | | | | | | | | | | | | | | | | |
| --- | --- | --- | --- | --- | --- | --- | --- | --- | --- | --- | --- | --- | --- | --- | --- | --- | --- | --- | --- |
|  | Elias (survivor) | Elias (non-survivor) | Jabbari (survivor) | Jabbari (non-survivor) | Jabbari *P* values | De Carvalho (survivor) | De Carvalho (non-survivor) | Barman (survivor) | Barman (non-survivor) | Barman *P* values | Sarvelloni (survivor) | Sarvelloni (non-survivor) | Sarvelloni *P* values | Dengeri (survivor) | Dengeri (non-survivor) | Dengeri *P* values | Kunt (survivor) | Kunt (non-survivor) | Kunt *P* values |
| Sinus rhythm, n (%) | 537 (79) | 20 (47) | 176 (94.1) | 49 (86) | 0.006 | 211 (89) | 28 (12) |  |  |  | 172 (90.5) | 16 (66.7) | <0.0001 | 133 (83.6) | 7 (16.4) | <0.001 |  |  |  |
| Non-sinus rhythm |  |  | 6 (3.3) | 8 (14) |  |  |  |  |  |  |  |  |  |  |  |  |  |  |  |
| Sinus Bradycardia, n (%) | 13 (2) | 2 (5) |  |  |  |  |  |  |  |  |  |  |  |  |  |  |  |  |  |
| Normal axis, n (%) |  |  | 147 (80.8) | 40 (70.2) | 0.136 |  |  |  |  |  |  |  |  |  |  |  |  |  |  |
| Left axis deviation, n (%) |  |  | 30 (16.5) | 16  (28.1) |  |  |  |  |  |  |  |  |  |  |  |  |  |  |  |
| Right axis deviation, n (%) |  |  | 5 (2.7) | 1 (1.8) |  |  |  |  |  |  |  |  |  |  |  |  |  |  |  |
| Abnormal axis, n (%) |  |  |  |  |  | 11 (4) | 5 (14) |  |  |  |  |  |  |  |  |  |  |  |  |
| Atrial Ectopy, n (%) | 59 (9) | 7 (18) |  |  |  |  |  |  |  |  |  |  |  |  |  |  |  |  |  |
| Sinus Tachycardia, n (%) | 207 (31) | 13 (30) |  |  |  |  |  | 70 (27) | 29 (43) | 0.008 |  |  |  |  |  |  |  |  |  |
| Atrial Fibrillation or atrial flutter, n (%) | 34 (5) | 8 (19) |  |  |  |  |  |  |  |  | 10 (6) | 8 (33.3) | <0.0001 | 6 (3.6) | 14 (32.3) | <0.001 | 5 (1.4) | 3 (4.2) | <0.001 |
| Other arrhythmias (Savelloni et al., defined this as sinus tachycardia and premature ventricular and supraventricular extrasystoles). |  |  |  |  |  |  |  |  |  |  | 13 (7.8) | 5 (20.8) | 0.057 |  |  |  |  |  |  |
| Left Atrial Enlargement, n (%) |  |  |  |  |  | 2 (1) | 1 (3) |  |  |  |  |  |  |  |  |  |  |  |  |
| Left Ventricular Hypertrophy, n (%) | 86 (13) | 10 (24) |  |  |  | 3 (1) | 0 |  |  |  |  |  |  |  |  |  |  |  |  |
| Pacemaker rhythm, n (%) |  |  |  |  |  | 3 (1) | 3 (8) |  |  |  |  |  |  |  |  |  |  |  |  |
| Pathological Q wave, n (%) | 63 (9) | 9 (21) |  |  |  | 29 (12) | 4 (11) |  |  |  |  |  |  |  |  |  |  |  |  |
| Any repolarisation abnormality, n (%) |  |  |  |  |  | 64 (27) | 8 (22) |  |  |  |  |  |  |  |  |  |  |  |  |
| Giant T wave, n (%) |  |  |  |  |  | 14 (6) | 0 |  |  |  |  |  |  |  |  |  |  |  |  |
| Pathological negative T wave, n (%) |  |  | 20 (11) | 8 (14) |  | 52 (22) | 7 (19) | 73 (28) | 31 (47) | 0.004 |  |  |  | 24 (15.3) | 1 (3.3) | 0.066 | 11 (3.2) | 1 (1.4) |  |
| ST segment depression, n (%) |  |  | 14 (7.6) | 0 |  | 15 (6) | 1 (3) |  |  |  |  |  |  |  |  |  | 2 (0.6) | 2 (2.8) | <0.001 |
| ST segment depression >1mm, n (%) |  |  |  |  |  |  |  | 63 (24) | 31 (47) | <0.001 |  |  |  |  |  |  |  |  |  |
| Any ST depression or elevation ≥1mm, n (%) | 38 (6) | 2 (5) |  |  |  |  |  |  |  |  |  |  |  |  |  |  |  |  |  |
| Any ST depression or elevation, n (%) | 104 (15) | 14 (33) |  |  |  |  |  |  |  |  |  |  |  |  |  |  |  |  |  |
| ST segment elevation V1, n (%) |  |  |  |  |  |  |  | 12 (4) | 9 (13) | 0.008 |  |  |  |  |  |  |  |  |  |
| ST segment elevation, n (%) |  |  | 10 (5.5) | 6 (10.5) | 0.223 | 6 (3) | 0 |  |  |  |  |  |  | 3 (1.8) | 1 (3.3) | 0.518 |  |  |  |
| Atrioventricular block |  |  |  |  |  |  |  |  |  |  | 15 (9) | 5 (20.8) | 0.081 |  |  |  |  |  |  |
| First degree atrioventricular block (PR >240ms), n (%) | 14 (2) | 0 | 0 | 1 (1.8) |  | 14 (6) | 0 |  |  |  |  |  |  | 9 (5.4) | 1 (3.3) | 0.524 |  |  |  |
| Second degree atrioventricular block, n (%) |  |  | 2 (1.1) | 0 |  |  |  |  |  |  |  |  |  |  |  |  |  |  |  |
| Left posterior hemiblock |  |  |  |  |  |  |  |  |  |  | 2 (1.2) | 0 | 1 |  |  |  |  |  |  |
| Left anterior hemiblock, n (%) |  |  |  |  |  | 8 (3) | 3 (8) |  |  |  | 28 (19.8) | 9 (37.5) | 0.0258 |  |  |  |  |  |  |
| Left vetricular hypertrophy, n (%) |  |  | 13 (7.1) | 4 (7) |  | 3 (1) | 0 |  |  |  |  |  |  |  |  |  | 4 (1.2) | 2 (2.8) | <0.001 |
| Right vetricular hypertrophy, n (%) |  |  | 1 (0.5) | 0 |  |  |  |  |  |  |  |  |  |  |  |  |  |  |  |
| Delta wave, n (%) |  |  |  |  |  | 2 (1) | 0 |  |  |  |  |  |  |  |  |  |  |  |  |
| Intraventicular block, n (%) |  |  |  |  |  | 32 (12) | 11 (30) |  |  |  |  |  |  |  |  |  |  |  |  |
| Low QRS voltage | 25 (4) | 4 (10) |  |  |  |  |  |  |  |  |  |  |  |  |  |  |  |  |  |
| QRS >120ms, n (%) | 65 (10) | 5 (12) |  |  |  |  |  |  |  |  |  |  |  |  |  |  |  |  |  |
| Incomplete right bundle branch block, n (%) |  |  |  |  |  |  |  | 21 (8) | 14 (21) | 0.002 |  |  |  |  |  |  |  |  |  |
| Right bundle branch block, n (%) |  |  | 6 (3.3) | 0 |  | 11 (5) | 2 (5) | 16 (6) | 10 (15) | 0.017 | 18 (10.8) | 4 (16.7) | 0.407 | 16 (9.9) | 7 (16.1) | 0.25 | 11 (3.2) | 2 (2.8) |  |
| Left bundle branch block, n (%) |  |  | 2 (0.8) | 1 (0.5) |  | 6 (3) | 5 (14) | 7 (2) | 2 (3) | 0.889 | 4 (2.4) | 2 (8.3) | 0.122 | 14 (9) | 8 (19.4) | 0.102 | 3 (0.9) | 4 (5.6) | <0.001 |
| Ventricular extrasystoles, n (%) |  |  | 9 (4.9) | 3 (5.3) | 1 | 11 (5) | 0 |  |  |  |  |  |  |  |  |  |  |  |  |
| U wave, n (%) |  |  |  |  |  | 14 (6) | 1 (3) |  |  |  |  |  |  |  |  |  |  |  |  |
| Poor R wave progression, n (%) | 109 (16) | 9 (21) |  |  |  | 8 (3) | 0 |  |  |  |  |  |  |  |  |  |  |  |  |
| Prolonged QTc | 55 (8) | 9 (21) |  |  |  |  |  |  |  |  | 39 (23.49) | 16 (66.6) | <0.0001 | 77 (48.6) | 32 (75) | <0.001 |  |  |  |
| Right ventricular strain, n (%) | 33 (5) | 1 (2.4) |  |  |  |  |  | 59 (22) | 36 (54) | <0.001 |  |  |  |  |  |  | 14 (4) | 2 (2.8) |  |
| S1Q3T3 Pattern, n (%) |  |  | 7 (3.8) | 0 |  |  |  | 33 (12) | 17 (25) | 0.009 | 18 (10.8) | 7 (29.1) | 0.013 |  |  |  |  |  |  |
| S1, n (%) |  |  |  |  |  |  |  |  |  |  | 30 (18) | 9 (37.5) | 0.562 |  |  |  |  |  |  |
| Q3, n (%) |  |  |  |  |  |  |  |  |  |  | 45 (27.1) | 15 (62.5) | <0.0001 |  |  |  |  |  |  |
| T3, n (%) |  |  |  |  |  |  |  |  |  |  |  |  |  |  |  |  |  |  |  |
| P Pulmonale, n (%) |  |  |  |  |  |  |  |  |  |  |  |  |  |  |  |  |  |  |  |
| CLOCKROT (clockwise rotation of QRS vector in precordial leads, n (%) |  |  |  |  |  |  |  | 45 (17) | 21 (31) | 0.01 |  |  |  |  |  |  |  |  |  |
| Negative T wave V2, n (%) |  |  |  |  |  |  |  | 29 (11) | 13 (19) | 0.063 |  |  |  |  |  |  |  |  |  |
| QR in V1, n(%) |  |  |  |  |  |  |  | 17 (6) | 8 (12) | 0.133 |  |  |  |  |  |  |  |  |  |
| Key (percentages given as % of patients within group (survived or died) with specified rhythm | | | | | | | | | | | | | | | | | | | |

Table S5. Full statistically significant (P<0.05) results used in data synthesis

| **ECG finding** | **Included study (survivors, non-survivors and P-values)** | | | | | | | | | | | | | | | |
| --- | --- | --- | --- | --- | --- | --- | --- | --- | --- | --- | --- | --- | --- | --- | --- | --- |
|  | Jabbari (survivors) | Jabbari (non-survivors) | Jabbari *P* values | Barman (survivors) | Barman (non-survivors) | Barman *P* values | Sarvelloni (survivors) | | Sarvelloni (non-survivors) | Sarvelloni *P* values | Dengeri (survivors) | Dengeri (non-survivors) | Dengeri *P* values | Kunt (survivor) | Kunt (non-survivor) | Kunt *P* values |
| Sinus rhythm, n (%) | 176 (94.1) | 49 (86) | 0.01 |  |  |  | 172 (90.5) | | 16 (66.7) | <0.0001 | 133 (83.6) | 7 (16.4) | <0.001 |  |  |  |
| Sinus Tachycardia, n (%) |  |  |  | 70 (27) | 29 (43) | 0.008 |  | |  |  |  |  |  |  |  |  |
| Atrial Fibrillation or atrial flutter, n (%) |  |  |  |  |  |  | 10 (6) | | 8 (33.3) | <0.0001 | 6 (3.6) | 14 (32.3) | <0.001 | 5 (1.4) | 3 (4.2) | <0.001 |
| Pathological negative T wave, n (%) |  |  |  | 73 (28) | 31 (47) | 0.004 |  | |  |  |  |  |  |  |  |  |
| ST segment depression, n (%) |  |  |  |  |  |  |  | |  |  |  |  |  | 2 (0.6) | 2 (2.8) | <0.001 |
| ST segment depression >1mm, n (%) |  |  |  | 63 (24) | 31 (47) | <0.001 |  | |  |  |  |  |  |  |  |  |
| ST segment elevation V1, n (%) |  |  |  | 12 (4) | 9 (13) | 0.008 |  | |  |  |  |  |  |  |  |  |
| Left anterior hemiblock, n (%) |  |  |  |  |  |  | 28 (19.8) | | 9 (37.5) | 0.0258 |  |  |  |  |  |  |
| Left bundle branch block, n (%) |  |  |  |  |  |  |  | |  |  |  |  |  | 3 (0.9) | 4 (5.6) | <0.001 |
| Incomplete right bundle branch block, n (%) |  |  |  | 21 (8) | 14 (21) | 0.002 |  | |  |  |  |  |  |  |  |  |
| Right bundle branch block, n (%) |  |  |  | 16 (6) | 10 (15) | 0.017 |  | |  |  |  |  |  |  |  |  |
| Prolonged QTc |  |  |  |  |  |  | 39 (23.49) | | 16 (66.6) | <0.0001 | 77 (48.6) | 32 (75) | <0.001 |  |  |  |
| Right ventricular strain, n (%) |  |  |  | 59 (22) | 36 (54) | <0.001 | |  |  |  |  |  |  |  |  |  |
| S1Q3T3 Pattern, n (%) |  |  |  | 33 (12) | 17 (25) | 0.009 | 18 (10.8) | | 7 (29.1) | 0.013 |  |  |  |  |  |  |
| Q3, n (%) |  |  |  |  |  |  |  | |  |  |  |  |  |  |  |  |
| T3, n (%) |  |  |  |  |  |  | 45 (27.1) | | 15 (62.5) | <0.0001 |  |  |  |  |  |  |
| P Pulmonale, n (%) |  |  |  |  |  |  |  | |  |  |  |  |  |  |  |  |
| CLOCKROT (clockwise rotation of QRS vector in precordial leads, n (%) |  |  |  | 45 (17) | 21 (31) | 0.01 |  | |  |  |  |  |  |  |  |  |
| Negative T wave V2, n (%) |  |  |  | 29 (11) | 13 (19) | 0.063 |  | |  |  |  |  |  |  |  |  |
| Left ventricular hypertrophy, n (%) |  |  |  |  |  |  |  | |  |  |  |  |  | 4 (1.2) | 2 (2.8) | <0.001 |
| Key (percentages given as % of patients within group (survived or died) with specified rhythm | | | | | | | | | | | | | | | | |

Table S6. Statistically significant ECG changes and their association with mortality as a percentage of the included populations.

|  | Jabbari (survived | Jabbari (died) | Barman (survived) | Barman (died) | Sarvelloni (survived) | Sarvelloni (died) | Dengeri (survived) | Dengeri (died) |
| --- | --- | --- | --- | --- | --- | --- | --- | --- |
| Sinus rhythm, n (%) | 94.1 | 86 |  |  | 90.5 | 66.7 | 83.6 | 16.4 |
| Sinus Tachycardia, n (%) |  |  | 27 | 43 |  |  |  |  |
| Atrial Fibrillation or atrial flutter, n (%) |  |  |  |  | 6 | 33.3 | 3.6 | 32.3 |
| Pathological negative T wave, n (%) |  |  | 28 | 47 |  |  |  |  |
| ST segment depression >1mm, n (%) |  |  | 24 | 47 |  |  |  |  |
| ST segment elevation V1, n (%) |  |  | 4 | 13 |  |  |  |  |
| Left anterior hemiblock, n (%) |  |  |  |  | 19.8 | 37.5 |  |  |
| Incomplete right bundle branch block, n (%) |  |  | 8 | 21 |  |  |  |  |
| Right bundle branch block, n (%) |  |  | 6 | 15 |  |  |  |  |
| Prolonged QTc |  |  |  |  | 23.49 | 66.6 | 48.6 | 75 |
| Right ventricular strain, n (%) |  |  | 22 | 54 |  |  |  |  |
| S1Q3T3 Pattern, n (%) |  |  | 12 | 25 | 10.8 | 29.1 |  |  |
| Q3, n (%) |  |  |  |  | 27.1 | 62.5 |  |  |
| CLOCKROT (clockwise rotation of QRS vector in precordial leads, n (%) |  |  | 17 | 31 |  |  |  |  |
| Negative T wave V2, n (%) |  |  | 11 | 19 |  |  |  |  |
|  |  |  |  |  |  |  |  |  |
| Key (percentages given as % of patients within group (survived or died) with defined rhythm |  |  |  |  |  |  |  |  |

Table S7. Statistically significant ECG changes and their association with mortality as a percentage of the included populations. Data used to formulate Figure 2.

|  | Barman *et al*., (2021) | Sarvelloni *et al.,* (2022) | Dengeri *et al.*, (2021) | Kunt et al., (2021) |
| --- | --- | --- | --- | --- |
| Sinus Tachycardia | 43 |  |  |  |
| Atrial Fibrillation or atrial flutter |  | 33.3 | 32.3 | 4.2 |
| Pathological negative T wave | 47 |  |  |  |
| ST segment depression |  |  |  | 2.8 |
| ST segment depression >1mm | 47 |  |  |  |
| ST segment elevation V1 | 13 |  |  |  |
| Left anterior hemiblock |  | 37.5 |  |  |
| Incomplete right bundle branch block | 21 |  |  |  |
| Right bundle branch block | 15 |  |  |  |
| Left bundle branch block |  |  |  | 5.6 |
| Prolonged QTc |  | 66.6 | 75 |  |
| Right ventricular strain | 54 |  |  |  |
| S1Q3T3 Pattern | 25 | 29.1 |  |  |
| T3 |  | 62.5 |  |  |
| CLOCKROT | 31 |  |  |  |
| Negative T wave V2 | 19 |  |  |  |
| Left ventricular hypertrophy |  |  |  | 2.8 |

Jabbari excluded as only data available for sinus rhythm

Table S8. Sinus rhythm prevalence as a percentage among survivors compared with non-survivors. Data used to formulate Figure 4.

|  | Jabbari (survivor) | Jabbari (non-survivor) | De Carvalho (survivor) | De Carvalho (non-survivor) | Sarvelloni (survivor) | Sarvelloni (non-survivor) | Dengeri (survivor) | Dengeri (non-survivor) |
| --- | --- | --- | --- | --- | --- | --- | --- | --- |
| Sinus rhythm, n (%) | 94.1 | 86 | 89 | 12 | 90.5 | 66.7 | 83.6 | 16.4 |

| **ECG change** | **Elias (aOR)** | **Jabbari (aOR)** | **De Carvalho (aOR)** | **Raad (aOR)** | **Barman (aOR)** | **Salvelloni* (aHR)** |
| --- | --- | --- | --- | --- | --- | --- |
| Atrial flutter or Atrial fibrillation | 2.54 (1.05-6.2) *P* 0.39 |  |  |  |  |  |
| HR >100bpm | 1.3 (0.88-1.93) *P* 0.194 | 1.015 (1.021-1.862) *P* 0.185 |  |  |  |  |
| Left bundle branch block |  |  | 7.1 (1.9-25.1) ***P* 0.002** |  |  |  |
| ST segment abnormality | 2.38 (1.49-3.84) ***P* <0.001** |  |  |  |  |  |
| Non-sinus rhythm |  | 7.961 (1.724-36.759) ***P* 0.008** |  |  |  |  |
| ST elevation |  |  |  |  |  |  |
| Axis deviation (abnormal axis) |  |  | 3.9 (1.1-11.5) ***P* 0.02** |  |  |  |
| ST depression |  |  |  |  |  |  |
| Fragmented QRS |  |  |  |  |  |  |
| Right ventricular strain | 2.7 (1.30-6.12) ***P* 0.007** |  |  | 15.2 (5.1-45.2) ***P* <0.001** | 4.385 (2.226–8.638) ***P* < 0.001** | 2.94 (1.01-8.55), ***P* 0.047** |
| QTc interval >451ms |  |  |  |  |  | 3.24 (1.09-9.62) ***P* 0.033** |
| T p-e / QTc >0.20 |  |  |  |  |  | 0.79 (0.28-2.20) *P* 0.662 |

Table S9. Adjusted Odds Ratios (aOR) and Adjusted Hazard Ratios (aHR) (Statistically significant P values in bold)

Savelloni *et al.* (2021) produced three statistically significant adjusted HRs which are displayed within this table but not included within the Forest plot to increase clinical homogeneity of the Forest Plot.
