# Supplementary material for: Are Pre‐Hospitalization ECG Abnormalities Associated With Increased Mortality in COVID‐19 Patients? A Quantitative Systematic Literature Review
Source: Ann Noninvasive Electrocardiol. 2024 Oct 12;29(6):e70016. doi: 10.1111/anec.70016 (PMC11470194; doi:10.1111/anec.70016)
Supplement: Supplementary file 1 — Appendix S1. [file ANEC-29-e70016-s001.zip › anec70016-sup-0003-Supinfo2.docx]

Supplementary information 2. ECG changes and prevalence in survivors compared with non-survivors (*P* values included where available)
