# Supplementary material for: Are Pre‐Hospitalization ECG Abnormalities Associated With Increased Mortality in COVID‐19 Patients? A Quantitative Systematic Literature Review
Source: Ann Noninvasive Electrocardiol. 2024 Oct 12;29(6):e70016. doi: 10.1111/anec.70016 (PMC11470194; doi:10.1111/anec.70016)
Supplement: Supplementary file 1 — Appendix S1. [file ANEC-29-e70016-s001.zip › anec70016-sup-0004-Supinfo3.docx]

Supplementary information 3. Adjusted Odds Ratios and Hazard Ratios.

| **ECG change** | **Elias** | **Jabbari** | **De Carvalho** | **Raad** | **Barman** | **Salvelloni (adjusted hazard ratios)** |
| --- | --- | --- | --- | --- | --- | --- |
| Atrial flutter or Atrial fibrillation | 2.54 (1.05-6.2) *P* 0.39 |  |  |  |  |  |
| HR >100bpm | 1.3 (0.88-1.93) *P* 0.194 | 1.015 (1.021-1.862) *P* 0.185 |  |  |  |  |
| Left bundle branch block |  |  | 7.1 (1.9-25.1) ***P* 0.002** |  |  |  |
| ST segment abnormality | 2.38 (1.49-3.84) ***P* <0.001** |  |  |  |  |  |
| Non-sinus rhythm |  | 7.961 (1.724-36.759) ***P* 0.008** |  |  |  |  |
| ST elevation |  |  |  |  |  |  |
| Axis deviation (abnormal axis) |  |  | 3.9 (1.1-11.5) ***P* 0.02** |  |  |  |
| ST depression |  |  |  |  |  |  |
| Fragmented QRS |  |  |  |  |  |  |
| Right ventricular strain | 2.7 (1.30-6.12) ***P* 0.007** |  |  | 15.2 (5.1-45.2) ***P* <0.001** | 4.385 (2.226–8.638) ***P* < 0.001** | 2.94 (1.01-8.55), ***P* 0.047** |
| QTc interval >451ms |  |  |  |  |  | 3.24 (1.09-9.62) ***P* 0.033** |
| T p-e / QTc >0.20 |  |  |  |  |  | 0.79 (0.28-2.20) *P* 0.662 |

Adjusted HRs for Salvelloni

Statistically significant P values in bold
